# Successful Use of Kidneys from a Deceased Donor with Active Herpes Zoster Infection

**DOI:** 10.1155/2021/7719041

**Published:** 2021-08-16

**Authors:** Amanda J. Vinson, Prakash Chauhan, Christopher Daley, Himanthi De Silva, Karthik K. Tennankore, Paul Bonnar, Kenneth A. West

**Affiliations:** ^1^Division of Nephrology, Department of Medicine, Nova Scotia Health, Halifax, NS, Canada; ^2^Division of General Surgery, Department of Surgery, Nova Scotia Health, Halifax, NS, Canada; ^3^Multi-Organ Transplant Program, Nova Scotia Health, Halifax, NS, Canada; ^4^Division of Infectious Disease, Department of Medicine, Nova Scotia Health, Halifax, NS, Canada

## Abstract

**Background:**

The limited donor pool and increasing recipient wait list require a reevaluation of kidney organ suitability for transplantation. Use of higher infectious risk organs that were previously discarded may help improve access to transplantation and reduce patient mortality without placing patients at a higher risk of poor posttransplant outcomes. There is very little data available regarding the safe use of kidney organs from deceased donors with varicella zoster virus infection at the time of organ retrieval. *Case Presentation*. Here, we report a case of successful transplantation of both kidneys from a deceased donor with active herpes zoster infection at the time of organ retrieval. Recipients were treated preemptively with acyclovir. At 4 months posttransplant, both kidney recipients experienced no infectious complications and were off dialysis with functioning transplant grafts.

**Conclusions:**

The use of kidney organs from donors with active herpes zoster infection appears to be a safe option to expand the kidney donor pool.

## 1. Background

Given the limited donor pool and increasing demand for organs, there has been an incentive to utilize higher risk organs for transplantation, including those from donors with increased infectious risk [1]. Following transplantation, recipients require lifelong maintenance immunosuppression to prevent acute rejection and graft loss, thereby placing these patients at increased risk for opportunistic or donor-transmitted infections.

Varicella zoster virus (VZV) is an alpha-herpesvirus with seropositivity in approximately 90% of adults in the United States [2]. It presents clinically as two distinct forms. In patients with primary disease (varicella or chickenpox), the virus is disseminated, and following resolution, VZV establishes lifelong latency in cranial and dorsal root ganglia. VZV may also undergo reactivation in a seropositive patient resulting in herpes zoster (HZ) (shingles). The latter is characterized by painful dermatomal vesicular eruptions which may rarely become disseminated.

HZ infection in transplant recipients is a significant cause of morbidity and in rare cases may be fatal [3]. The incidence of HZ infection in solid organ transplant (SOT) recipients may be 10 to 100 times higher than in the immunocompetent general population [4]. Most HZ infection in SOT recipients is hypothesized to represent reactivation of latent recipient-derived virus. However, the role of donor-derived VZV infection is less well understood. There is one prior case report of a pediatric cardiac transplant recipient who was seronegative for VZV and developed donor-transmitted VZV infection from a donor treated for severe primary varicella 10 days prior [5]. A second case of suspected donor-derived VZV infection following lung transplantation was reported from a seropositive VZV donor in a recipient who was also VZV seropositive pretransplant [6]. Given the uncertainty around the risk of potential donor-derived VZV infection, whether or not to accept kidney organs from donors who have active VZV infection at the time of retrieval is unknown.

There are no reports to date regarding the use of kidney organs from donors with active HZ infection at the time of donation. We describe the first case of successful transplantation of two kidneys from a deceased donor with active VZV shingles infection at the time of organ retrieval.

## 2. Case Presentation

An older patient with severe anoxic brain injury after cardiac arrest became a donor after cardiac death. Prior to donation, the donor was noted to have a three-day history of a vesicular erythematous rash on the left flank extending into the left groin. The donor was noted to be positive for VZV IgG serology, and a swab of a vesicular lesion was positive for VZV. The infectious disease team was consulted for the donor and diagnosed active HZ infection without evidence of dissemination or immunosuppression, for which the donor received 2 days of treatment dose intravenous valacyclovir, the last dose being given the morning of organ retrieval. On collateral history, there were no preceding symptoms of encephalitis. The donor was negative for cytomegalovirus (CMV).

After a comprehensive discussion regarding potential donor-derived infection risks between the transplant nephrologist, transplant surgeon, and infectious disease team, kidneys were allocated to two recipients on dialysis, both of whom were VZV immune. The first was a 52-year-old female with panel reactive antibody (PRA) 0%, 3 human leukocyte antigen (HLA) mismatch (MM) with the donor, CMV negative, and Epstein Barr virus (EBV) immune. The second recipient was a 51-year-old female with PRA 50%, 6 HLA MM, CMV positive, and EBV immune. Both had end-stage kidney disease secondary to type 1 diabetes mellitus and were induced with Basiliximab. Maintenance immunosuppression consisted of mycophenolate mofetil (MMF) 1 g PO bid, prednisone 5 mg daily, and tacrolimus to target a trough level of 8-10 in the first 3 months, then 5-8 thereafter. Both patients received perioperative antibiotic prophylaxis with cephalexin and prophylaxis with oral sulfamethoxazole 400 mg/trimethoprim 80 mg (for urinary tract infections and *Pneumocystis jirovecii*). According to our local protocol, neither patient received CMV prophylaxis. Given active HZ in the donor, both patients received treatment dose acyclovir 10 mg/kg IV on POD 0, then were transitioned to renally adjusted treatment dose oral acyclovir for 7 days (800 mg five times daily, corrected for estimated GFR) followed by prophylactic renally dosed oral acyclovir (400 mg daily or bid based on changes in estimated GFR over time) until 3 months posttransplant. Recipients were monitored clinically for symptoms and signs of VZV infection.

Both patients had a slow improvement in graft function and required one session of dialysis for hyperkalemia and fluid overload in the first week posttransplant. Otherwise, outside of issues with nausea and vomiting attributed to diabetic gastroparesis, there were no perioperative complications. Both patients had nuclear renal scans which demonstrated good flow but features consistent with acute tubular necrosis. By the second week posttransplant, the creatinine was beginning to fall for both recipients. Neither patient had any infectious complications relating to VZV infection, although Recipient 1 did develop BK viremia at 3 months posttransplant that responded to reduction of her immunosuppression. At the last follow-up 4 months posttransplant, both recipients remained well. Creatinine for Recipients 1 and 2 was 124 and 198, respectively.

## 3. Discussion

Herein, we report for the first time the successful utilization of both kidneys from a deceased donor with confirmed active, nondisseminated HZ infection at the time of donation. The recipients were both VZV immune prior to transplant and received Basiliximab induction therapy with standard triple immunosuppression. They were treated prophylactically with high-dose acyclovir for a one-week period followed by prophylactic dose acyclovir for 3 months posttransplant. Both recipients did well with no evidence of VZV reactivation or infectious complications.

To our knowledge, only two prior cases of donor-derived VZV infection have been reported in the literature [5, 6]. The first case was in a pediatric cardiac transplant recipient who was VZV seronegative prior to transplant and received a heart from a donor with primary varicella infection treated 10 days prior. The patient received prophylaxis with acyclovir 200 mg intravenously tid for 6 days posttransplant and then was transitioned to oral dosing. On postoperative day 12, the patient developed a nonspecific macular, erythematous rash and was diagnosed with VZV infection. Acyclovir dose was increased to 400 mg IV five times daily, and immunosuppression was reduced with a complete and uncomplicated recovery. The second case was in a VZV seropositive lung recipient who received a lung transplant from a VZV seropositive donor and subsequently developed fatal septic shock [6]. A summary of our recipient cases and the two prior case reports of deceased donors with donor-derived VZV transmission at the time of organ retrieval is shown in Table 1.

In response to the limited donor pool and the growing number of patients on the transplant waiting list, there is a drive to accept more marginal and higher infectious risk organs. Thus, increased infectious risk donors presently make up approximately 20% of the deceased donor pool in the United States [7]. As an example, the use of Hepatitis C-infected donor kidneys for Hepatitis C-negative recipients has recently been trialed with direct acting antiviral agents administered either immediately posttransplant [8] or following detection of viremia in the recipient [9]. This has resulted in the transplantation of excellent quality donor organs that otherwise would have been discarded, with no adverse infectious outcomes reported.

Primary varicella infection is rare in adult SOT recipients but can be catastrophic when it occurs, presenting with severe skin or visceral involvement and disseminated intravascular coagulation [10]. Conversely, HZ is relatively common following transplantation in those with prior VZV infection or vaccination, with a reported incidence of 8-11% in the first 4 years posttransplant [10]. Postherpetic neuralgia also occurs with increased frequency (~20-40%) in the transplant population compared with the general population [11]. While invasive HZ disease is rare, it has been reported with fatal consequences in those with immunosuppressed status [10], and disseminated VZV infection in kidney transplant recipients has been associated with a 30% mortality rate [3].

The treatment of primary VZV infection posttransplant includes high-dose intravenous acyclovir, initiated early in the course of illness. There is also some evidence for reduction of immunosuppression. Conversely, cutaneous nondisseminated, nonfacial, or ophthalmologic HZ can be treated with oral acyclovir, valacyclovir, or famciclovir. Those patients with herpes ophthalmicus or Ramsay-Hunt syndrome (reactivation of VZV in the geniculate ganglion which may lead to facial palsy) and disseminated or organ invasive HZ should be treated with IV acyclovir, akin to primary varicella [10].

Pretransplant vaccination in seronegative patients and antiviral prophylaxis posttransplant with acyclovir or valganciclovir have dramatically reduced the incidence of VZV reactivation; however, the risk persists for many years posttransplant, and there is limited evidence on long-term prophylactic therapy. Given the risk of VZV reactivation in the posttransplant period, the recombinant subunit vaccine Shingrix is recommended in transplant recipients over the age of 50 years.

In conclusion, the relative risk and benefit of accepting increased infectious risk donor organs including from donors with active HZ infection must be considered in the context of the increasing demand for donor organs and the corresponding risk of death or comorbidity accrual on the transplant waiting list if such organs are discarded. Our experience is the first documented report of utilizing organs from a donor with active HZ infection, and we demonstrate excellent outcomes with no complications or adverse infectious outcomes as a result.

## Figures and Tables

**Table 1:** Demographic data, clinical manifestations, treatment choices, and clinical course of recipients following transplantation with organs from donors with active VZV at organ retrieval.

ID	Age	Gender	Organ type	Recipient comorbidities	Recipient VZV serostatus	Donor presentation	Anti-VZV prophylaxis	Clinical outcome
Recipient 1	52 years	F	Kidney	Diabetes type 1	Immune	Active shingles	Acyclovir 10 mg/kg IV × 1 dose, then renally adjusted oral acyclovir equivalent to 800 mg PO 5 times/day^∗^ × 7 days, then 400 mg PO daily/bid^∗^ until 3 months posttransplant	No infectious complications at 4 months of follow-up
Recipient 2	51 years	F	Kidney	Diabetes type 1	Immune	Active shingles	Acyclovir 10 mg/kg IV × 1 dose, then renally adjusted oral acyclovir equivalent to 800 mg PO 5 times/day^∗^ × 7 days, then 400 mg PO daily/bid^∗^ until 3 months posttransplant	No infectious complications at 4 months of follow-up
Case 3 ^5^	15 months	F	Heart	Dilated cardiomyopathy due to endocardial fibroelastosis	Nonimmune	Severe chickenpox 10 days prior to donation	Acyclovir 200 mg IV tid × 6 days then 200 mg PO tid	Fever, macular rash, vesicles, VZV IgM day 12, VZV IgG month 4. Uncomplicated recovery
Case 4 ^6^	62 years	F	Lung	Diffuse bronchiectasis	Immune	VZV seropositive but no clinical varicella infection	None given prophylactically but acyclovir given when patient destabilized and identified to have VZV viremia	VZV reinfection and death

^∗^Acyclovir dosing adjusted based on evolving renal function over time.

## Data Availability

All data generated or analyzed during this study are included in this published article.

## Abbreviations

SOT: Solid organ transplant
HZ: Herpes zoster
VZV: Varicella zoster virus
CMV: Cytomegalovirus
PRA: Panel reactive antibody
HLA: Human leukocyte antigen
MMF: Mycophenolate mofetil.
